# Improved Glomerular Filtration Rate Estimation by an Artificial Neural Network

**DOI:** 10.1371/journal.pone.0058242

**Published:** 2013-03-13

**Authors:** Xun Liu, Xiaohua Pei, Ningshan Li, Yunong Zhang, Xiang Zhang, Jinxia Chen, Linsheng Lv, Huijuan Ma, Xiaoming Wu, Weihong Zhao, Tanqi Lou

**Affiliations:** 1 Division of Nephrology, Department of Internal Medicine, The Third Affiliated Hospital of Sun Yat-sen University, Guangzhou, China; 2 Department of Biomedical Engineering, South China University of Technology, Guangzhou, China; 3 Division of Nephrology, Department of Geriatrics, The First Affiliated Hospital of Nanjing Medical University, Nanjing, China; 4 Department of Radiation Oncology, Chengdu International Cancer Treatment Hospital, Chengdu, China; 5 School of Information Science & Technology, Sun Yat-sen University, Guangzhou, China; 6 Department of Internal Medicine, JieYang People's Hospital, Jieyang, China; 7 Operating Room, The Third Affiliated Hospital of Sun Yat-sen University, Guangzhou, China; University of Adelaide, Australia

## Abstract

**Background:**

Accurate evaluation of glomerular filtration rates (GFRs) is of critical importance in clinical practice. A previous study showed that models based on artificial neural networks (ANNs) could achieve a better performance than traditional equations. However, large-sample cross-sectional surveys have not resolved questions about ANN performance.

**Methods:**

A total of 1,180 patients that had chronic kidney disease (CKD) were enrolled in the development data set, the internal validation data set and the external validation data set. Additional 222 patients that were admitted to two independent institutions were externally validated. Several ANNs were constructed and finally a Back Propagation network optimized by a genetic algorithm (GABP network) was chosen as a superior model, which included six input variables; i.e., serum creatinine, serum urea nitrogen, age, height, weight and gender, and estimated GFR as the one output variable. Performance was then compared with the Cockcroft-Gault equation, the MDRD equations and the CKD-EPI equation.

**Results:**

In the external validation data set, Bland-Altman analysis demonstrated that the precision of the six-variable GABP network was the highest among all of the estimation models; i.e., 46.7 ml/min/1.73 m^2^ vs. a range from 71.3 to 101.7 ml/min/1.73 m^2^, allowing improvement in accuracy (15% accuracy, 49.0%; 30% accuracy, 75.1%; 50% accuracy, 90.5% [*P*<0.001 for all]) and CKD stage classification (misclassification rate of CKD stage, 32.4% vs. a range from 47.3% to 53.3% [*P*<0.001 for all]). Furthermore, in the additional external validation data set, precision and accuracy were improved by the six-variable GABP network.

**Conclusions:**

A new ANN model (the six-variable GABP network) for CKD patients was developed that could provide a simple, more accurate and reliable means for the estimation of GFR and stage of CKD than traditional equations. Further validations are needed to assess the ability of the ANN model in diverse populations.

## Introduction

Chronic kidney disease (CKD) is a major public health problem worldwide [1]. The Center for Disease Control in the USA declared that the prevalence of CKD was 26 million in the United States [2] and the number of patients with CKD in China was estimated to be about 119.5 million [3]. CKD is a serious threat to health and quality of life [4]. The number of patients that accepted maintenance renal replacement therapy in the United States increased from 281,000 in 2000 to 547,000 in 2010 to 571,000 in 2011 [5]. Currently, over 270,000 chronic hemodialysis patients were registered in the Chinese Renal Data System [6].

Accurate evaluation of glomerular filtration rates (GFRs) is of critical importance in clinical practice and research [7]. Although inulin clearance and renal radionuclide excretion rates are the gold standards to determine GFRs, they cannot be used widely because of inconvenience and high cost. Therefore, serum creatinine (SC)-based estimating equations for GFR were developed. The National Kidney Foundation - Kidney Disease Outcomes Quality Initiative Working Group recommended that the Cockcroft-Gault equation [8] and the Modification of Diet in Renal Disease (MDRD) equations [9] could be used to calculate the GFRs of adults [10]. In order to improve the accuracy of estimation, the MDRD researchers in 2006 used a more accurate isotope dilution mass spectrometry to measure the SC level, and they developed re-expressed MDRD formulas [11]. Furthermore, the studies were extended to 8,254 cases. The newly estimated GFR (eGFR) formula of the Chronic Kidney Disease Epidemiology Collaboration (CKD-EPI) equation was revised [12]. However, the correct CKD stage classification rates of the Cockcroft-Gault and MDRD formulas were only 64% and 62%, suggesting that the traditional SC-based equations remain relatively imprecise in the estimation of GFR [13] due to SC and the non-GFR determinants introducing significant errors when calculating the GFR [14]. Such imprecision can potentially result in misclassification of the CKD stage, which leads to both incorrect diagnosis and treatment for individuals and bias in estimating the prevalence of CKD in the general population [15]. Finding a more accurate method for estimating GFR is an urgent problem that needs to be solved.

Recently, Inker et al. developed a new estimating equation based on cystatin C in combination with creatinine and found that the combined equation performed better than equations based on either marker alone [13]. These results indicated that the combination of novel filtration markers, such as cystatin C and SC, into the GFR estimating formula may be a key factor for improving the accuracy of estimation. However, the incremental cost of introducing the new marker should be considered.

The traditional GFR estimation equations were all developed by the linear regression method. A large number of samples, a priori knowledge, and specific limits such as absence of multi-collinearity between independent variables were necessary during the development of the equations. With the development of modern mathematics and information technology, artificial neural networks (ANNs) are one of the methods of mathematical modeling that has been widely applied in the field of engineering prediction. An ANN has been applied in the field of medicine and biology as well, such as cardiac output [16] and in other physiological measurements [17]–[18]. A specifically trained three-layer ANN can infinitely approximate any linear or nonlinear function with precision [19]-[20]. Traditionally, the Back Propagation (BP) networks are widely used, though they have inherent defects [21]. More complicated ANN models have been recently published with greater descriptions of the construction of the models and software sharing [22]–[23]. A genetic algorithm, a random search algorithm enlightened from biological natural selection and genetic mechanisms, can be applied to optimize BP networks [24] for better performance.

In a previous study, we found that the Radial Basis Function network was superior to the traditional equations at estimating GFR [25]. In the large-sample cross-sectional survey reported here, we assessed the performance of a BP network optimized by a genetic algorithm (GABP network) for the estimation of GFR, which had similar features to the Radial Basis Function network.

## Methods

### Patients

Chronic kidney disease was defined and staged according to the National Kidney Foundation - Kidney Disease Outcomes Quality Initiative clinical practice guidelines [10]. Patients with acute kidney function deterioration, clinical edema, skeletal muscle atrophy, pleural effusion or ascites, malnutrition, amputation, heart failure or ketoacidosis were excluded from the study. Patients that were younger than 18 years were excluded. Patients that were taking cimetidine or trimethoprim were excluded as well. No subject was being treated with dialysis at the time of the study.

### Measurement

The GFR was measured by the method of technetium-99 m diethylenetriaminepentaacetic acid (^99 m^Tc-DTPA) renal dynamic imaging (modified Gate's method) was used as the standard GFR (sGFR) [24]-[25]. A Millennium TMMPR SPECT with a General Electric Medical System was used to measure ^99 m^Tc-DTPA renal dynamic imaging as previously described [26]. There was good agreement between ^99 m^Tc-DTPA renal imaging and plasma clearance of 51 chromium ethylenediamine tetraacetic acid [27]. An enzymatic method was used to measure SC. Values of SC in the development data set, the internal validation data set and the external validation data set were all traceable to the National Institute of Standards and Technology creatinine standard reference material (SRM 967). Data on gender, age, height, and weight were recorded at the same time.

### Study design

From January 2005 through December 2009, 831 patients with CKD in the third affiliated hospital of Sun Yat-sen University, China, were enrolled, of which 562 patients were randomly selected as the development data set and the remaining 269 patients constituted the internal validation data set. From January 2010 through December 2010, 349 patients in the same hospital were included in the external validation data set. An additional 222 patients were admitted to two independent institutions in other Chinese cities for external validation (Table 1 and Table S1). Stages 1 and 2, as well as stages 4 and 5 were combined for convenience. The study protocol was approved by the institutional review board at the Third Affiliated Hospital of Sun Yat-sen University and written informed consent was obtained before the study.

**Table 1:** Patient characteristic.

Characteristic	Development and Internal Validation (N = 831)	External Validation (n = 349)	Additional External Validation (n = 222)
Causes of CKD, N (%)			
Primary glomerular disease	255(30.7)	71(20.3)	71(32.0)
Diabetic nephropathy	205(24.0)	147(42.1)	48(21.6)
Hypertension	115(13.8)	44(12.6)	45(20.3)
Chronic tubulointerstitial disease	81(9.7)	30(8.6)	16(7.2)
Polycystic kidney disease	27(3.2)	8(2.3)	2(0.9)
Lupus nephritis	13(1.6)	5(1.4)	5(2.3)
Other causes or causes unknown	135(16.2)	44(12.6)	35(15.8)
Distribution of CKD stages, N (%)		‡	*
CKD 1	62(7.5)	32(9.2)	39(17.6)
CKD 2	167(20.1)	75(21.5)	63(28.4)
CKD 3	310(37.3)	140(40.1)	73(32.9)
CKD 4	195(23.5)	80(22.9)	32(14.4)
CKD 5	97(11.7)	22(6.3)	15(6.8)
Age, mean (s.d.) in years	53(17)	58(15)*	57(17)†
Male / Female (%)	63.4/36.6	60.2/39.8	54.1/45.9‡
Weight, mean (s.d.), kg	61(11)	62(12)	62(10)
Height, mean (s.d.), cm	163(8)	162(8)	164(7)
BMI, mean (s.d.), kg/m^2^	23(3)	23(4)‡	23(3)
BSA, mean (s.d.), m^2^	1.65(0.17)	1.66(0.18)	1.67(0.15)
Serum albumin, mean (s.d.), g/dL	3.8(0.7)	3.8(0.6)	3.9(0.7)‡
Serum urea nitrogen, mean (s.d.), mg/dL	37(24)	36(26)	30(23)*
Serum creatinine, mean (s.d.), mg/dL	3.0(2.7)	2.5(2.3)†	2.8(3.4)
sGFR, mean (s.d.), ml/min/1.73 m^2^	45 (27)	49 (27)‡	60(32)*

* :*P*<0.001 compared with the combined development and internal validation data sets.

† :*P*<0.01 compared with the combined development and internal validation data sets.

‡ :*P*<0.05 compared with the combined development and internal validation data sets.

Abbreviations: CKD, chronic kidney disease; BMI, body mass index; BSA, body-surface area; sGFR, standard glomerular filtration rate.

Independent variables taken into account included albumin (Alb), serum urea nitrogen (SUN), SC, age, height, weight and gender, and the only dependent variable was estimated GFR. Gender as a binary variable was transformed with dumb variable encoding; e.g., male equaled 1 and female equaled 0. As the range of each variable from the raw data was not the same, and it would affect construction of the ANN, each variable was normalized into the same range. The maximum and minimum values of normalization are shown in Table S2, and all minimum values were set to be not less than 0 considering the practical significance of the data.

### Modeling with the ANN

A three-layer BP network was constructed using commercial software (Matlab software version 2011b, The Mathworks, Boston MA, USA). The neurons of the input layer included all independent variables as the input variables of the network, and the neuron of the output layer was the dependent variable; i.e., eGFR, as the output variable of the network. Each neuron of the hidden layer took the S function as an exciting function, and several networks were constructed with different numbers of neurons in the hidden layer (1 to 13). Each BP network was initialized randomly and then trained by learning the rule of back propagation with the development data set, and was validated with the internal validation data set to achieve a superior topology. Performance was defined as mean square error of the internal validation data set. A set of thresholds and weights could be specified after training, and then the output of the network was calculated by the weighted summation of each neuron to approximate sGFR.

To achieve better performance of the ANN, initialization of the weights and thresholds of the BP network was optimized by the Genetic algorithm (GABP network). All weights and thresholds of one network were encoded as a chromosome, and then evolved from one generation to another, including the progression of mutation and crossing. When a network could achieve better performance in the internal validation data set, the initial weights and thresholds were selected for the next generation. Finally, superior initial weights and thresholds were achieved, and then applied in the initialization of the network.

To facilitate clinical use, we used a mean impact value analysis [28] to select variables from the seven input variables of the GABP network gradually and, in turn, excluded Alb, gender, height, SUN, weight and age. We then established the appropriate GABP network with different input variables. The six-variable (including SC, age, weight, SUN, height and gender) GABP network with a topology of 6-2-1 (named the GABP6 network) was the optimal model in the internal validation data set. Explanations of the network are listed in Tables S3, S4, S5, S6, S7, S8, S9, S10, S11, S12, S13, S14, S15 and Figure S1. Detailed performances in the internal validation data set are presented in Tables S16 and S17, and Figures S2 and S3.

### Calculations

The GFR was estimated by using the following equations:

1. Cockcroft-Gault-equation (CG) [8]: 
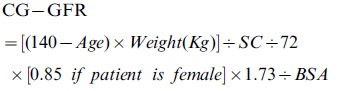

2. Six-variable MDRD equation (MDRD1) [9]:
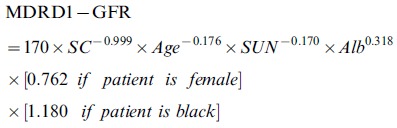

3. Four-variable MDRD equation (MDRD4) [9]:
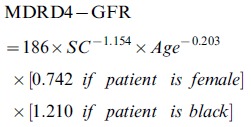

4. CKD-EPI equation (CKD-EPI) [12]:
  - κ = 0.7(Female)or 0.9(Male);
  - α = −0.329 (Female and SC≤0.7 mg/dl), α = −1.209(Female and SC>0.7 mg/dl);
  - α = −0.411(Male and SC≤0.9 mg/dl), α = −1.209(Male and SC>0.9 mg/dl)

### Statistical analysis

Quantitative data were expressed as mean ± SD or as median. The difference between eGFR and standard GFR (sGFR) was defined as eGFR minus sGFR. Accuracy was defined as the percentage of estimated GFR not deviating more than 15, 30, and 50% from the sGFR. The precision was defined as the width between the 95% limits of agreement. A prior acceptable tolerance for the precision was defined 60 ml/min/1.73 m^2^ [29]. The difference between eGFR and sGFR was regressed against the average of eGFR and sGFR. The bias for eGFR was expressed as the slope of the regression line against the X-axis. The trend of accuracy for eGFR was expressed as the intercept of the regression line against the Y-axis. Independent samples t-test was used to compare the quantitative variables between two data sets. Wilcoxon Mann-Whitney test and Pearson's chi-squared test were used to compare the difference and accuracy between two data sets. Wilcoxon signed rank test and McNemar test were used to compare the difference and accuracy within data set. ANCOVA tests were used to compare first the slopes, and then the intercepts of the regression line. All statistics were performed using SPSS software (version 11.0 SPSS, Chicago IL, USA) and Medcalc for Windows (version 9.3.9.0 Medcalc software, Mariekerke, Belgium).

## Results

### Patients

The clinical characteristics of the development data set (*n* = 562), internal validation data set (*n* = 269) external validation data set (*n* = 349) and the additional external validation data set (*n* = 222) are shown in Table 1 and Table S1. In the development data set, the mean sGFR was 46.1 ml/min/1.73 m^2^ (SD, 27.0 ml/min/1.73 m^2^) and ranged from 3.3 ml/min/1.73 m^2^ to 130.1 ml/min/1.73 m^2^. The external validation data set had a similar mean sGFR, gender, weight, height, body surface area (BSA) and mean SUN level with the development and internal validation data sets but differed in the distribution of CKD stages, age, body mass index (BMI), and mean Alb and SC levels.

### Performance of the estimation models in the external validation data set

**Figure 1:**
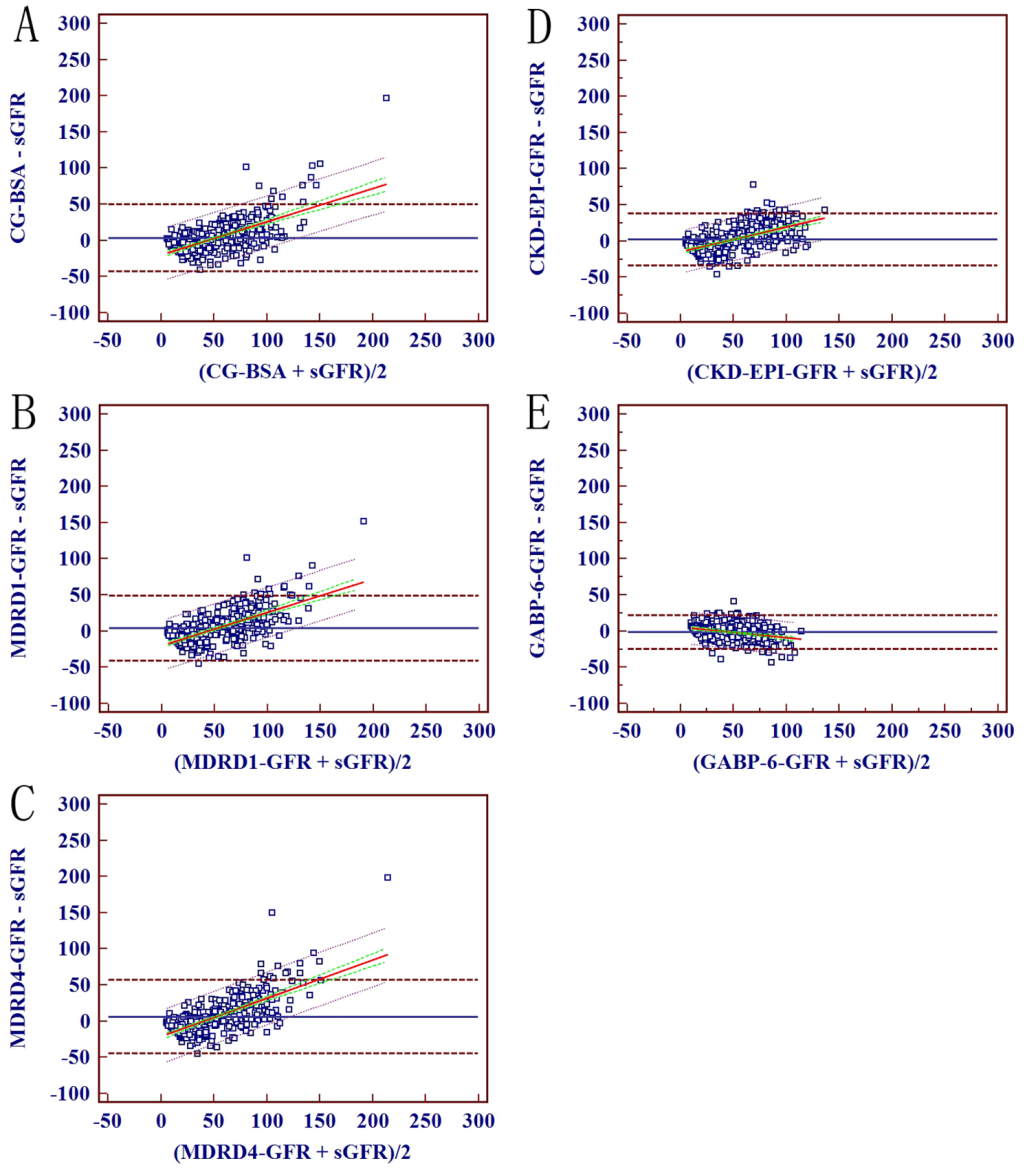
Bland–Altman plot of eGFR and sGFR (ml/min/1.73 m^2^) in the external validation data set. Solid blue line represents the mean of difference between methods; dashed brown lines represent 95% limits of agreement of the mean of difference between methods; solid red line represents the regression line of difference between methods against average of methods; dotted green lines represent 95% confidence intervals for the regression line, and dashed purple lines represent 95% limits of agreement of the regression line. A, B, C, D and E represent for the results of GFR estimated by the Cockcroft-Gault equation, the six variable MDRD equation, the four variable MDRD equation, the CKD-EPI equation and the six variable GABP network, respectively. Abbreviations: eGFR, estimated glomerular filtration rate; sGFR, standard glomerular filtration; CG, Cockcroft-Gault equation; MDRD: Modification of Diet in Renal Disease; CKD-EPI: Chronic Kidney Disease Epidemiology Collaboration; GABP: BP network with genetic algorithm.

Bland-Altman analysis demonstrated that the precision of the six-variable GABP network was the highest among all of the estimation models (46.7 ml/min/1.73 m^2^ vs. a range from 71.3 ml/min/1.73 m^2^ to 101.7 ml/min/1.73 m^2^). Therefore, we chose eGFR calculated by the six-variable GABP network as the reference against which all comparisons between estimation models were made. Both the slope and the intercept of the regression line of the six-variable GABP network were improved (slope, −0.15 ml/min/1.73 m^2^ vs. a range from 0.34 ml/min/1.73 m^2^ to 0.53 [*P*<0.001 for all]; intercept, 5.88 vs. a range from −14.79 to −21.54 [*P*<0.01 for all]; Table 2, Figure 1). The accuracies within 15%, 30% and 50% of the six-variable GABP network were all the greatest (*P*<0.001 for all), and the median percent of the absolute difference was least (15.61 ml/min/1.73 m^2^ vs. a range from 26.00 ml/min/1.73 m^2^ to 36.21 ml/min/1.73 m^2^, *P*<0.001 for all; Table 3).

**Table 2:** Overall performance of agreement between eGFR and sGFR in the external validation data set.

	Precision (ml/min/1.73 m^2^)	Slope of regression line with the X-axis^a^ (95% CI)	Intercepts of regression line with the Y-axis^a^ (95% CI)
CG equation	92.8	0.46(0.40,0.52)*	−19.83(−23.40,−16.26)†
MDRD1 equation	90.3	0.46(0.40,0.51)*	−19.44(−22.85,−16.02)*
MDRD4 equation	101.7	0.53(0.47,0.59)*	−21.54(−25.11,−17.64)*
CKD-EPI equation	71.3	0.34(0.29,0.39)*	−14.79(−17.78,−11.80)†
GABP6 network	46.7	−0.15(−0.20,−0.10)	5.88(3.20,8.55)

Abbreviations: eGFR, estimated glomerular filtration rate; sGFR, standard glomerular filtration; CG, Cockcroft-Gault; MDRD, Modification of Diet in Renal Disease; CKD-EPI, Chronic Kidney Disease Epidemiology Collaboration; GABP, BP network with genetic algorithm

a:The difference between eGFR and sGFR was regressed against the average of eGFR and sGFR. *X*-axis represented the average of eGFR and sGFR. *Y*-axis represented the difference between eGFR and sGFR. eGFR, estimated glomerular filtration rate; sGFR, standard glomerular filtration rate.

* :*P*<0.001 compared with GABP6 network-GFR.

† :*P*<0.01 compared with GABP6 network-GFR.

‡ :*P*<0.05 compared with GABP6 network-GFR.

**Table 3:** Overall performance of difference and accuracy between eGFR and sGFR in the external validation data set.

	Median of difference (25%, 75% Percentile)	Median % Absolute difference (25%, 75% Percentile)	Accuracy within		
			15%	30%	50%
CG equation	−1.23(9.96,12.25)	26.00(13.03,47.55)*	29.2*	55.0*	77.6*
MDRD1 equation	−0.70(−10.16,15.22)	31.71(13.75,52.25)*	26.3*	46.7*	72.2*
MDRD4 equation	1.18(−9.48,16.38)‡	32.21(14.08,54.45)*	26.9*	46.1*	70.7*
CKD-EPI equation	−0.12(−9.95,13.51)†	30.74(12.57,50.90)*	26.9*	49.6*	73.9*
GABP6 network	−0.26(−8.54,5.73)	15.61(8.44,29.87)	49.0	75.1	90.5

* :*P*<0.001 compared with GABP6 network-GFR.

† :*P*<0.01 compared with GABP6 network-GFR.

‡ :*P*<0.05 compared with GABP6 network-GFR.

Abbreviations: eGFR, estimated glomerular filtration rate; sGFR, standard glomerular filtration; CG: Cockcroft-Gault; MDRD: Modification of Diet in Renal Disease; CKD-EPI: Chronic Kidney Disease Epidemiology Collaboration; GABP: BP network with genetic algorithm

The performance of the six-variable GABP network in various stages of CKD was analyzed. The median of the difference of the six-variable GABP network was less than the traditional equations in CKD stages 1–2 and CKD stages 4–5 (*P*<0.001 for all), as was the absolute difference in CKD stages 1–2 and CKD stage 3 (*P*<0.001 for all). Accuracy within 30% and 50% of the six-variable GABP network in CKD stages 1–2 and CKD stage 3 were the greatest (*P*<0.001 for all). There was also improvement in accuracy within 15% of the six-variable GABP network in CKD stages 1–2 (*P*<0.001 for all). All estimation models showed the same variation trend for performances from CKD stage 1 to CKD stage 5; that is, performance of one specific CKD stage became worse with the progression of CKD stage. This is due to sGFR becoming smaller during the progression of CKD stages, and therefore the relative error becoming greater. Detailed performances are listed in Table S18. We also evaluated misclassification of CKD by various estimation models. Misclassification for the diagnosis of moderate renal failure (GFR <60 ml/min/1.73 m^2^) as well as severe renal failure (GFR <15 ml/min/1.73 m^2^) were improved (*P*<0.01 for all) by the means of the six-variable GABP network (8.2% and 7.4%), as compared with those of the traditional equations (ranging from 12.6% to 13.2% and from 12.6% to 17.5%; Table 4). The six-variable GABP network improved the CKD stage misclassification rate (32.4% vs. a range from 47.3% to 53.3%, *P*<0.001 for all). In CKD stage 1 classified by various estimation models, the correct classification ratio of CKD stage 1 of the six-variable GABP network was significantly higher than for all traditional equations (90.9% vs. a range from 36.2% to 42.4%, *P*<0.01 for all). There were also some improvements in the correct classification ratios of the six-variable GABP network in CKD stage 2, CKD stage 4 as well as CKD stage 5, but without statistical significance (Table S19).

**Table 4:** CKD Misclassification in the external validation data set.

	Misclassification rate for the diagnosis of		CKD stage misclassification rate
	sGFR <60 ml/min/1.73 m^2^	sGFR <15 ml/min/1.73 m^2^	
CG equation	12.6†	12.6†	47.3*
MDRD1 equation	12.6†	17.2*	52.4*
MDRD4 equation	13.2†	17.5*	51.9*
CKD-EPI equation	12.9†	17.5*	53.3*
GABP6 network	8.3	7.4	32.4

* :*P*<0.001 compared with GABP6 network-GFR.

† :*P*<0.01 compared with GABP6 network-GFR.

‡ :*P*<0.05 compared with GABP6 network-GFR.

Abbreviations: sGFR, standard glomerular filtration rate; CG, Cockcroft-Gault; MDRD, Modification of Diet in Renal Disease; CKD-EPI, Chronic Kidney Disease Epidemiology Collaboration; GABP, BP network with genetic algorithm; CKD, chronic kidney disease

### Performance of the estimation models in the additional external validation data set

Bland-Altman analysis demonstrated that the precision of the six-variable GABP network was the highest among all of the estimation models (62.4 ml/min/1.73 m^2^ vs. a range from 68.0 ml/min/1.73 m^2^ to 73.5 ml/min/1.73 m^2^). The intercept of the regression line of the six-variable GABP network was improved (4.91 vs. a range from −16.07 to −18.05, *P*<0.01 for all). However, the slope of the regression line of the six-variable GABP network was the worst (−0.27 vs. a range from 0.18 to 0.24, *P*<0.001 for all; Table 5 and Figure S4), as was bias (median difference, −8.84 ml/min/1.73 m^2^vs. a range from −4.60 ml/min/1.73 m^2^ to −6.56 ml/min/1.73 m^2^; *P*<0.05 for all). The accuracies within 30% and 50% of the six-variable GABP network were all the greatest, and the median percent of the absolute difference was the least (20.75 ml/min/1.73 m^2^ vs. a range from 21.52 ml/min/1.73 m^2^ to 23.57 ml/min/1.73 m^2^, *P*<0.05 for all; Table 6). The misclassification rate for the diagnosis of severe renal failure (GFR <15 ml/min/1.73 m^2^) was also improved (11.3% vs. a range from 16.7% to 17.1%, *P*<0.01 for all) with the six-variable GABP network (Table 7).

**Table 5:** Overall performance of agreement between eGFR and sGFR in the additional external validation data set.

	Precision (ml/min/1.73 m^2^)	Slope of regression line with the X-axis^a^ (95% CI)	Intercepts of regression line with the Y-axis^a^ (95% CI)
CG equation	72.5	0.21(0.15,0.28)*	−17.99(−22.29,−13.69)†
MDRD1 equation	68.0	0.20(0.14,0.26)*	−16.16(−20.23,−12.09)*
MDRD4 equation	73.5	0.24(0.18,0.30)*	−18.05(−22.28,−13.83)*
CKD-EPI equation	68.4	0.18(0.12,0.25)*	−16.07(−20.24,−11.91)†
GABP6 network	62.4	−0.27(−0.34,−0.20)	4.91(0.76,9.06)

Abbreviations: eGFR, estimated glomerular filtration rate; sGFR, standard glomerular filtration rate; CG, Cockcroft-Gault; MDRD, Modification of Diet in Renal Disease; CKD-EPI, Chronic Kidney Disease Epidemiology Collaboration; GABP, BP network with genetic algorithm

a:The difference between eGFR and sGFR was regressed against the average of eGFR and sGFR. *X*-axis represented the average of eGFR and sGFR. *Y*-axis represented the difference between eGFR and sGFR.

* :*P*<0.001 compared with GABP6 network-GFR.

† :*P*<0.01 compared with GABP6 network-GFR.

‡ :*P*<0.05 compared with GABP6 network-GFR.

**Table 6:** Overall performance of difference and accuracy between eGFR and sGFR in the additional external validation data set.

	Median of difference (25%, 75% Percentile)	Median % Absolute difference (25%, 75% Percentile)	Accuracy within		
			15%	30%	50%
CG equation	−6.56(−16.85,3.42)‡	23.57(10.49,43.11)†	34.6	61.2	80.8†
MDRD1 equation	−4.60(−15.38,5.14)*	21.52(9.78,44.38)‡	39.2	63.3	78.3*
MDRD4 equation	−4.92(−15.02,5.10)*	23.26(8.94,46.84)†	34.2	60.4	76.7*
CKD-EPI equation	−5.71(−16.47,4.48)*	23.52(8.82,47.21)†	35.8	60.0*	77.1*
GABP6 network	−8.44(−19.57,0.22)	20.75(11.19,34.18)	34.6	67.5	88.8

Abbreviations: eGFR, estimated glomerular filtration rate; sGFR, standard glomerular filtration; CG: Cockcroft-Gault; MDRD: Modification of Diet in Renal Disease; CKD-EPI: Chronic Kidney Disease Epidemiology Collaboration; GABP: BP network with genetic algorithm

* :*P*<0.001 compared with GABP6 network-GFR.

† :*P*<0.01 compared with GABP6 network-GFR.

‡ :*P*<0.05 compared with GABP6 network-GFR.

**Table 7:** CKD Misclassification in the additional external validation data set.

	Misclassification rate for the diagnosis of		CKD stage misclassification rate
	sGFR <60 ml/min/1.73 m^2^	sGFR <15 ml/min/1.73 m^2^	
CG equation	9.0	16.7†	47.7
MDRD1 equation	10.4	16.7†	49.5
MDRD4 equation	10.4	17.1*	51.4‡
CKD-EPI equation	10.4	17.1*	53.6†
GABP6 network	9.5	11.3	42.3

Abbreviations: CG, Cockcroft-Gault; MDRD, Modification of Diet in Renal Disease; CKD-EPI, Chronic Kidney Disease Epidemiology Collaboration; GABP, BP network with genetic algorithm; CKD, chronic kidney disease

* :*P*<0.001 compared with GABP6 network-GFR.

† :*P*<0.01 compared with GABP6 network-GFR.

‡ :*P*<0.05 compared with GABP6 network-GFR.

According to the comprehensive information from the internal validation data set and both external validation data sets, the six-variable GABP network was selected as the optimal estimation model for patients with CKD. In order to show the model and facilitate external validations, a table based on the Excel software (File S1) was developed for convenience.

## Discussion

The GFR is defined as the number of milliliters of plasma per unit time from kidney filtration and is a direct indicator of glomerular filtration function. GFR is the basis of CKD definition and staging and it affects evaluation of evolution, prognosis and follow-up [7]. With a worsening baseline of renal function, patients seem to have a greater probability of progressing to a worse CKD stage in the next year [30]. Early detection and diagnosis are important means of effective prevention and treatment of CKD and its associated complications. Accurate evaluation of GFR is essential for CKD patients. Using this new ANN model (the six-variable GABP network, with a topology of 6-2-1), better precision and accuracy were achieved, which resulted in more accurate classification of severe renal failure (GFR <15 ml/min/1.73 m^2^). This will be of great help to physicians in making proper decisions for patients with CKD, thereby avoiding unnecessary diagnostic and therapeutic interventions. The previous finding [25] that the ANN was superior to the traditional equation in GFR estimation was supported as well by data. In conjunction with other studies [31]–[34], it indicated that the method of ANN may have an advantage in solving clinical problems.

In the field of medical data processing, the traditional statistical regression method takes the ‘law of large numbers’ as the theoretical basis, with some assumptions and prior knowledge. An equation is developed by collecting large amounts of data to fit the general law of the population. This equation is very dependent on the samples collected, which are supposed to have the same distribution as the population, so a decline in accuracy would happen when applied to the other population. In addition, the regression methods can only fit limited functional forms. Multicollinearity and interactions between independent variables also limit the application of regression methods. However, ANN, as a common method of machine learning, is widely applied in the fields of not only science and engineering but also medicine with its own advantages such as nonlinear mapping and robustness. This method does not require any a priori knowledge of the data. Multicollinearity and interaction is no longer a limitation of the application of this method. Even if the sample size is small, the law of population can still be learned from the sample with limited accuracy.

There were limitations in this study. First, SC in the MDRD equations [9] was measured by using the picric acid method. In the CKD-EPI equation [12] and the development data set, the internal validation data set and the external validation data set of our models, SC was determined by the enzymatic method traceable to isotope dilution-mass spectrometry. In the additional external validation data set of our models, SC levels were measured by the enzymatic method. The Cockcroft-Gault-equation [8] was developed long ago, and the methods of SC measurement are not available now. The difference in calibration of SC assays introduces error in the comparison between different GFR estimation models and subgroups [35]. Second, different estimation models used different ways to measure sGFR, which was also a source of system bias. Both the MDRD equations [9] and the CKD-EPI equation [12] used urinary clearances of ^125^I-iothalamate as the sGFR. In the Cockcroft-Gault equation [8], the method of sGFR measurement used the means of two 24-hour urine creatinine clearances. In this study, according to other studies [29], [36], sGFR was measured by the ^99m^Tc-DTPA renal dynamic imaging method. It is likely that differences in the results of our study and others were partly due to the use of different methods. Third, the sample contained only Chinese CKD patients. Further validations in separate studies with different races/ethnicities of CKD patients are needed to confirm the advantages of this ANN. Fourth, an ANN model is a ‘black box’, and cannot be expressed by a single mathematical equation. As a result, physicians are reluctant to accept the ANN's interpretation of data. In order to facilitate the application on a daily bedside basis, a simple table based on Excel software (File S1) was developed.

## Conclusions

A new ANN model (the six-variable GABP network) for CKD patients was developed and can provide a simple, more accurate and reliable means for the estimation of GFR and stage of CKD than traditional equations. Further validations are needed to assess the ability of ANN model in diverse populations.

## Supporting Information

Figure S1 **Topology of artificial neural network.**
(DOC)

Figure S2 **Bland–Altman plot of eGFR and sGFR (ml/min/1.73 m^2^) in the internal validation data set.** Solid blue line represents the mean of difference between methods; dashed brown lines represent 95% limits of agreement of the mean of difference between methods; solid red line represents the regression line of difference between methods against average of methods; dotted green lines represent 95% confidence intervals for the regression line, and dashed purple lines represent 95% limits of agreement of the regression line. A, B, C, D, E and F represent for the results of GFR estimated by GABP-7 network, GABP-6 network, GABP-5 network, GABP-4 network, GABP-3 network and GABP-2 network, respectively.
(DOC)

Figure S3 **Bland–Altman plot of eGFR and sGFR (ml/min/1.73 m^2^) in the internal validation data set.** Solid blue line represents the mean of difference between methods; dashed brown lines represent 95% limits of agreement of the mean of difference between methods; solid red line represents the regression line of difference between methods against average of methods; dotted green lines represent 95% confidence intervals for the regression line, and dashed purple lines represent 95% limits of agreement of the regression line. G represent for the results of GFR estimated by GABP-1 network.
(DOC)

Figure S4 **Bland–Altman plot of eGFR and sGFR (ml/min/1.73 m^2^) in the additional external validation data set.** Dotted blue line represents the mean of difference between methods; dashed brown lines represent 95% limits of agreement of the mean of difference between methods; solid red line represents the regression line of difference between methods against average of methods. A, B, C, D and E represent for the results of GFR estimated by the Cockcroft-Gault-equation, the six variable MDRD equation, the four variable MDRD equation, the CKD-EPI equation and the six variable GABP network, respectively.
(DOC)

Table S1 **Detailed characteristic in different subgroup of patients.**
(DOC)

Table S2 **Maximum and minimum values of normalization of raw data.**
(DOC)

Table S3 **Performance of GABP network with different topology.**
(DOC)

Table S4 **MIV analysis based on GABP network with a topology of 7-11-1.**
(DOC)

Table S5 **Performance of GABP network with 6 input variables.**
(DOC)

Table S6 **MIV analysis based on GABP network with a topology of 6-2-1.**
(DOC)

Table S7 **Performance of GABP network with 5 input variables.**
(DOC)

Table S8 **MIV analysis based on GABP network with a topology of 5-4-1.**
(DOC)

Table S9 **Performance of GABP network with 4 input variables.**
(DOC)

Table S10 **MIV analysis based on GABP network with a topology of 4-2-1.**
(DOC)

Table S11 **Performance of GABP network with 3 input variables.**
(DOC)

Table S12 **MIV analysis based on GABP network with a topology of 3-4-1.**
(DOC)

Table S13 **Performance of GABP network with 2 input variables.**
(DOC)

Table S14 **MIV analysis based on GABP network with a topology of 2-3-1.**
(DOC)

Table S15 **Performance of GABP network with 1 input variable.**
(DOC)

Table S16 **Overall performance of agreement between eGFR and sGFR in GABP networks with different input variables in the internal validation data set.**
(DOC)

Table S17 **Overall performance of difference and accuracy between eGFR and sGFR in GABP networks with different number of input variables in the internal validation data set.**
(DOC)

Table S18 **Performances between eGFR and sGFR in different stages of CKD in the external validation data set.**
(DOC)

Table S19 **Classification the CKD stage by the estimation models in different stages of CKD.**
(DOC)

File S1 **An Excel table based on the six-variable GABP network to estimate GFR.**
(XLS)
