# Supplementary material for: Improved Glomerular Filtration Rate Estimation by an Artificial Neural Network
Source: PLoS One. 2013 Mar 13;8(3):e58242. doi: 10.1371/journal.pone.0058242 (PMC3596400; doi:10.1371/journal.pone.0058242)
Supplement: Figure S1 — Topology of artificial neural network. (DOC) [file pone.0058242.s001.doc]

Figure S1. Topology of artificial neural network
