# Supplementary material for: Improved Glomerular Filtration Rate Estimation by an Artificial Neural Network
Source: PLoS One. 2013 Mar 13;8(3):e58242. doi: 10.1371/journal.pone.0058242 (PMC3596400; doi:10.1371/journal.pone.0058242)
Supplement: Figure S2 — Bland–Altman plot of eGFR and sGFR (ml/min/1.73 m2) in the internal validation data set. Solid blue line represents the mean of difference between methods; dashed brown lines represent 95% limits of agreement of the mean of difference between methods; solid red line represents the regression line of difference between methods against average of methods; dotted green lines represent 95% confidence intervals for the regression line, and dashed purple lines represent 95% limits of agreement of the regression line. A, B, C, D, E and F represent for the results of GFR estimated by GABP-7 network, GABP-6 network, GABP-5 network, GABP-4 network, GABP-3 network and GABP-2 network, respectively. (DOC) [file pone.0058242.s002.doc]

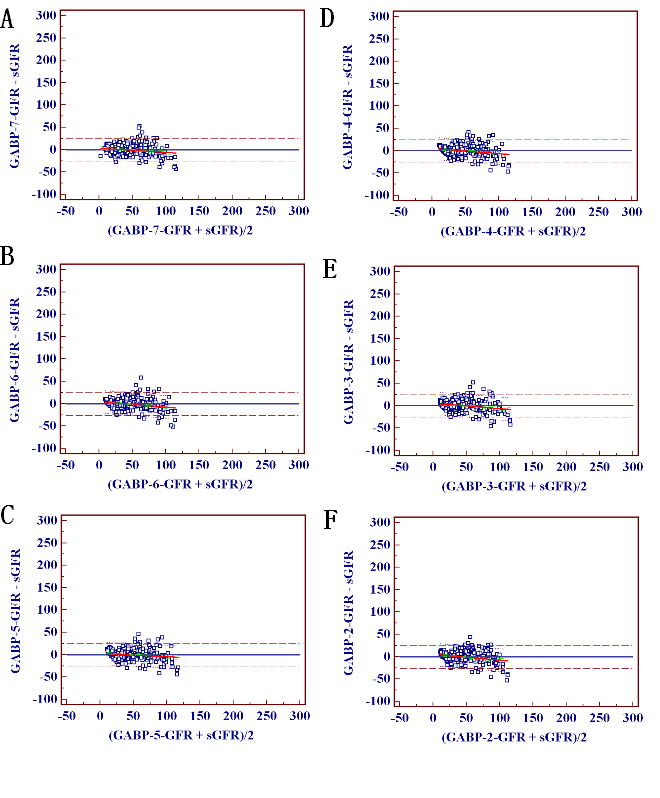


Figure S2. Bland–Altman plot of eGFR and sGFR (ml/min/1.73 m^2^) in the internal validation data set. Solid blue line represents the mean of difference between methods; dashed brown lines represent 95% limits of agreement of the mean of difference between methods; solid red line represents the regression line of difference between methods against average of methods; dotted green lines represent 95% confidence intervals for the regression line, and dashed purple lines represent 95% limits of agreement of the regression line. A, B, C, D, E and F represent for the results of GFR estimated by GABP-7 network, GABP-6 network, GABP-5 network, GABP-4 network, GABP-3 network and GABP-2 network, respectively.
