# Supplementary material for: Improved Glomerular Filtration Rate Estimation by an Artificial Neural Network
Source: PLoS One. 2013 Mar 13;8(3):e58242. doi: 10.1371/journal.pone.0058242 (PMC3596400; doi:10.1371/journal.pone.0058242)
Supplement: Figure S4 — Bland–Altman plot of eGFR and sGFR (ml/min/1.73 m2) in the additional external validation data set. Dotted blue line represents the mean of difference between methods; dashed brown lines represent 95% limits of agreement of the mean of difference between methods; solid red line represents the regression line of difference between methods against average of methods. A, B, C, D and E represent for the results of GFR estimated by the Cockcroft-Gault-equation, the six variable MDRD equation, the four variable MDRD equation, the CKD-EPI equation and the six variable GABP network, respectively. (DOC) [file pone.0058242.s004.doc]

**A**


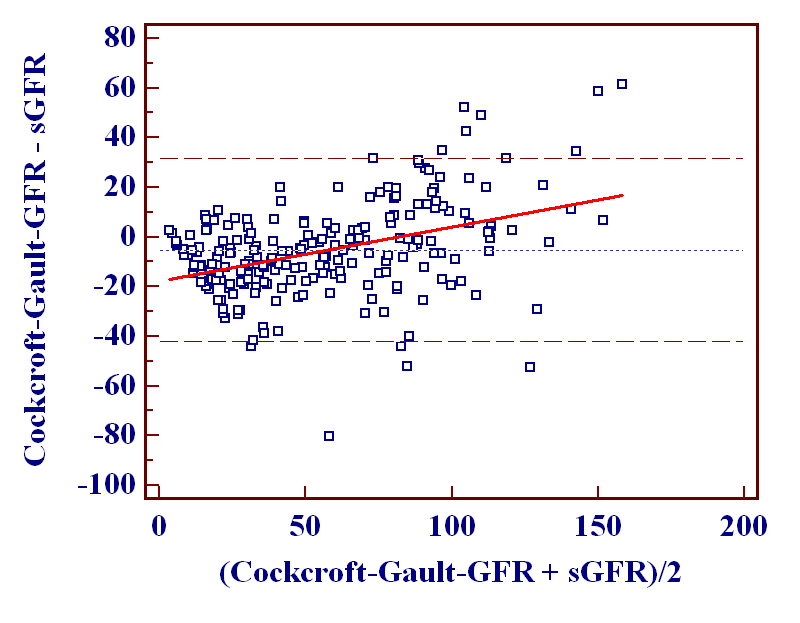


**B**


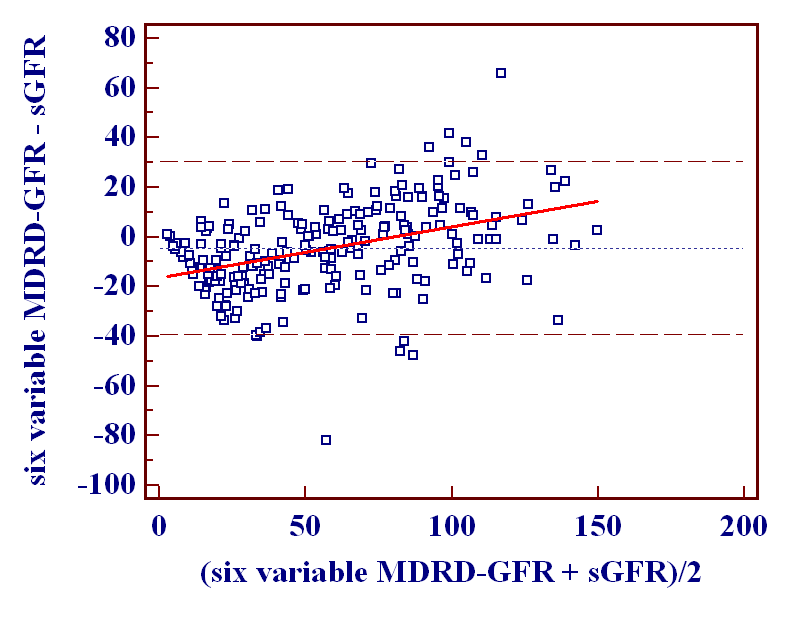


**C**

**
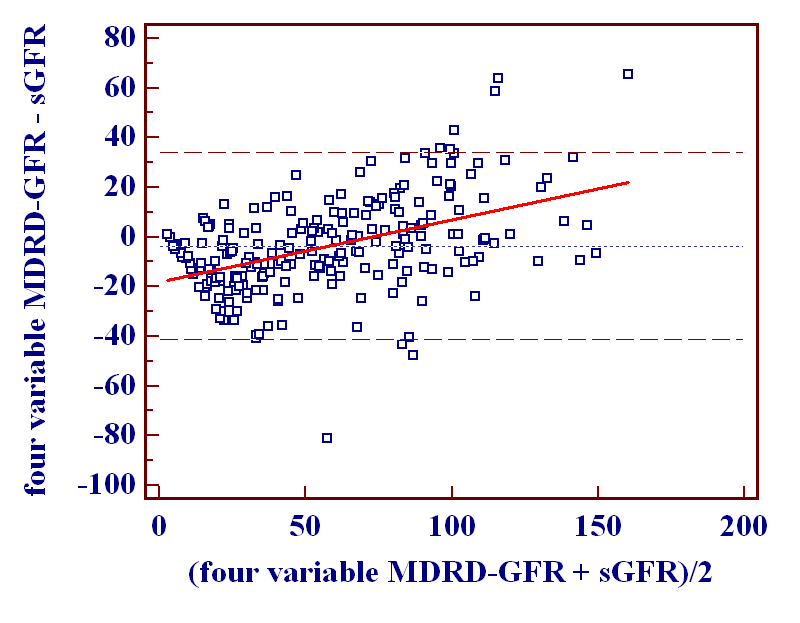
**

**D**

**
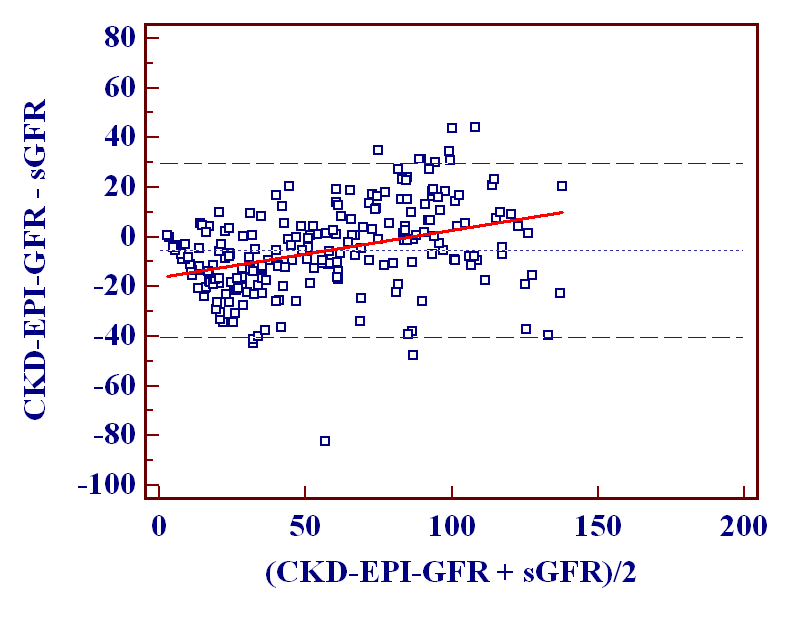
**

**E**

**
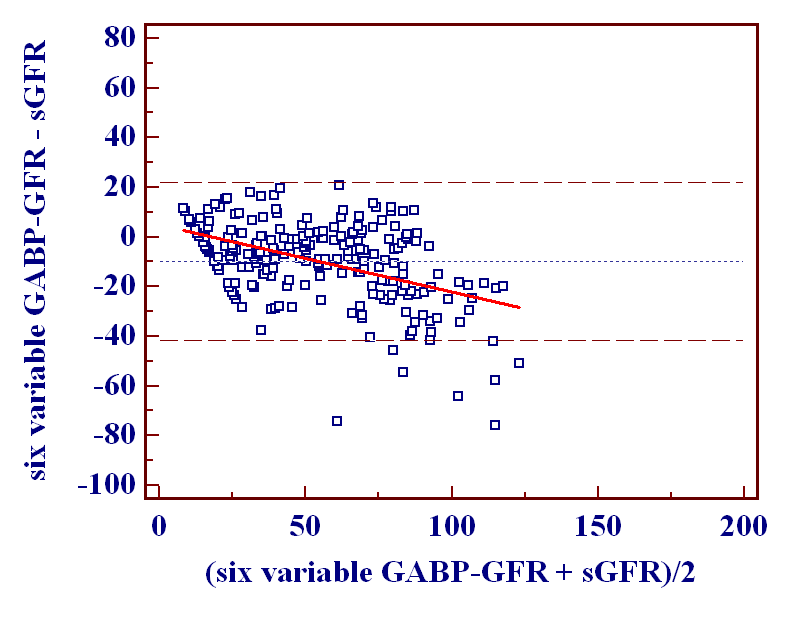
**

Figure S4. Bland–Altman plot of eGFR and sGFR (ml/min/1.73 m^2^) in the additional external validation data set. Dotted blue line represents the mean of difference between methods; dashed brown lines represent 95% limits of agreement of the mean of difference between methods; solid red line represents the regression line of difference between methods against average of methods. A, B, C, D and E represent for the results of GFR estimated by the Cockcroft-Gault-equation, the six variable MDRD equation, the four variable MDRD equation, the CKD-EPI equation and the six variable GABP network, respectively.
