# Supplementary material for: Improved Glomerular Filtration Rate Estimation by an Artificial Neural Network
Source: PLoS One. 2013 Mar 13;8(3):e58242. doi: 10.1371/journal.pone.0058242 (PMC3596400; doi:10.1371/journal.pone.0058242)
Supplement: Table S1 — Detailed characteristic in different subgroup of patients. (DOC) [file pone.0058242.s005.doc]

Table S1. Detailed characteristic in different subgroup of patients

| Characteristic | Development Data Set (n=562) | Internal Validation  Data Set (n=269) | External Validation  (n=349) | Additional External Validation-Jieyang Subgroup  (n=75) | Additional External Validation-Nanjing Subgroup  (n=147) |
| --- | --- | --- | --- | --- | --- |
| Causes of CKD |  |  |  |  |  |
| Primary glomerular disease | 163(29.0) | 92(34.2) | 71(20.3) | 46(61.3) | 25(17.0) |
| Diabetic nephropathy | 114(25.6) | 61(22.7) | 147(42.1) | 19(25.3) | 29(19.7) |
| Hypertension | 82(14.6) | 33(12.3) | 44(12.6) | 4(5.3) | 41(27.9) |
| Chronic tubulointerstitial disease | 57(10.1) | 24(8.9) | 30(8.6) | 0(0.0) | 16(10.9) |
| Polycystic kidney disease | 18(3.2) | 9(3.3) | 8(2.3) | 0(0.0) | 2(1.4) |
| Lupus nephritis | 6(1.1) | 7(2.6) | 5(1.4) | 5(6.7) | 0(0.0) |
| Other causes or causes unknown | 92(16.4) | 43(16.0) | 44(12.6) | 1(1.3) | 34(23.1) |
| Distribution of CKD stages |  |  |  |  |  |
| CKD 1 | 40(7.1) | 22(8.2) | 32(9.2) | 15(20.0) | 24(16.3) |
| CKD 2 | 122(21.7) | 45(16.7) | 75(21.5) | 8(10.7) | 55(37.4) |
| CKD 3 | 209(37.2) | 101(37.5) | 140(40.1) | 20(26.7) | 53(36.1) |
| CKD 4 | 131(23.3) | 64(23.8) | 80(22.9) | 20(26.7) | 12(8.2) |
| CKD 5 | 60(10.7) | 37(13.8) | 22(6.3) | 12(16.0) | 3(2.0) |
| Age (year) | 53(17) | 52(16) | 58(15) | 52(16) | 59(16) |
| Male / Female (%) | 63.3/36.7 | 63.6/36.4 | 60.2/39.8 | 61.3/38.7 | 61.1/38.9 |
| Weight (kg) | 61(11) | 61(11) | 62(12) | 59(13) | 63(9) |
| Height (cm) | 163(8) | 163(8) | 162(8) | 163(5) | 165(7) |
| BMI (kg/m^2^) | 23(3) | 23(4) | 23(4) | 22(4) | 23(3) |
| BSA (m^2^) | 1.65(0.17) | 1.66(0.17) | 1.66(0.18) | 1.63(0.17) | 1.69(0.13) |
| Serum albumin (g/dL) | 3.8(0.6) | 3.7(0.7) | 3.8(0.6) | 3.6(0.6) | 4.0(0.7) |
| Serum urea nitrogen (mg/dL) | 36(24) | 38(25) | 36(26) | 43(33) | 24(13) |
| Serum creatinine (mg/dL) | 2.9(2.6) | 3.2(2.9) | 2.5(2.3) | 5.4(4.7) | 1.0(1.0) |
| sGFR(ml/min/1.73m^2^) | 46(27) | 44(28) | 49 (27) | 52(41) | 64(26) |

Abbreviations: CKD, chronic kidney disease; BMI, body mass index; BSA, body-surface area; sGFR, standard glomerular filtration rate.
