# Supplementary material for: Improved Glomerular Filtration Rate Estimation by an Artificial Neural Network
Source: PLoS One. 2013 Mar 13;8(3):e58242. doi: 10.1371/journal.pone.0058242 (PMC3596400; doi:10.1371/journal.pone.0058242)
Supplement: Table S2 — Maximum and minimum values of normalization of raw data. (DOC) [file pone.0058242.s006.doc]

Table S2. Maximum and minimum values of normalization of raw data^*^

| variable | Minimum value | Maximum value |
| --- | --- | --- |
| Glomerular filtration rate | 0 | 166 |
| Age | 18 | 110 |
| Serum creatinine | 0 | 22 |
| Serum urea nitrogen | 0 | 241 |
| Serum albumin | 1 | 6 |
| Height | 134 | 193 |
| Weight | 18 | 110 |

*: Method of normalization: $y=\frac{x-x_{\min}}{x_{\max}-x_{\min}}$

Xmin means the minimum value and Xmax means the maximum value. The range of data is [0, 1] after normalization.
