# Supplementary material for: Improved Glomerular Filtration Rate Estimation by an Artificial Neural Network
Source: PLoS One. 2013 Mar 13;8(3):e58242. doi: 10.1371/journal.pone.0058242 (PMC3596400; doi:10.1371/journal.pone.0058242)
Supplement: Table S3 — Performance of GABP network with different topology. (DOC) [file pone.0058242.s007.doc]

Table S3. Performance of GABP network with different topology^*^

| Topology | Encoding length | MSE of development data | MSE of internal validation data |
| --- | --- | --- | --- |
| 7-1-1 | 10 | 179.0141 | 172.3869 |
| 7-2-1 | 19 | 172.3333 | 178.2567 |
| 7-3-1 | 28 | 171.4167 | 172.9514 |
| 7-4-1 | 37 | 176.8677 | 169.5948 |
| 7-5-1 | 46 | 177.0228 | 170.6669 |
| 7-6-1 | 55 | 174.8021 | 170.2407 |
| 7-7-1 | 64 | 167.0401 | 177.8274 |
| 7-8-1 | 73 | 171.8593 | 174.5800 |
| 7-9-1 | 82 | 168.1765 | 173.2617 |
| 7-10-1 | 91 | 165.0018 | 176.6264 |
| 7-11-1 | 100 | 175.3899 | 167.2615 |
| 7-12-1 | 109 | 171.0796 | 174.5672 |
| 7-13-1 | 118 | 172.2364 | 171.1981 |

*: When the topology is 7-11-1, a superior performance could be achieved.

Abbreviations: GABP, BP network with genetic algorithm; MSE, mean square error
