# Supplementary material for: Improved Glomerular Filtration Rate Estimation by an Artificial Neural Network
Source: PLoS One. 2013 Mar 13;8(3):e58242. doi: 10.1371/journal.pone.0058242 (PMC3596400; doi:10.1371/journal.pone.0058242)
Supplement: Table S4 — MIV analysis based on GABP network with a topology of 7-11-1. (DOC) [file pone.0058242.s008.doc]

Table S4. MIV analysis based on GABP network with a topology of 7-11-1^*^

| Input variable | MIV value | Rank |
| --- | --- | --- |
| Gender | -0.0438 | 1 |
| Serum creatinine | -0.0298 | 2 |
| Age | -0.0136 | 3 |
| Weight | -0.0105 | 4 |
| Height | -0.0045 | 5 |
| Serum urea nitrogen | -0.0023 | 6 |
| Serum albumin | 0.0016 | 7 |

*: Input variable Alb has minimum value of MIV, which indicates Alb has the least contribution to estimating dependent variable GFR, so a new GABP network could be constructed without Alb.

Abbreviations: GABP, BP network with genetic algorithm; MIV, mean impact value
