# Supplementary material for: Improved Glomerular Filtration Rate Estimation by an Artificial Neural Network
Source: PLoS One. 2013 Mar 13;8(3):e58242. doi: 10.1371/journal.pone.0058242 (PMC3596400; doi:10.1371/journal.pone.0058242)
Supplement: Table S5 — Performance of GABP network with 6 input variables. (DOC) [file pone.0058242.s009.doc]

Table S5. Performance of GABP network with 6 input variables^*^

| Topology | Encoding length | MSE of development data | MSE of internal validation data |
| --- | --- | --- | --- |
| 6-1-1 | 9 | 178.2136 | 175.2824 |
| 6-2-1 | 17 | 175.8132 | 170.0986 |
| 6-3-1 | 25 | 172.3864 | 173.3921 |
| 6-4-1 | 33 | 170.4500 | 175.7489 |
| 6-5-1 | 41 | 172.7828 | 172.9523 |
| 6-6-1 | 49 | 160.1758 | 181.5057 |

*: When the topology is 6-2-1, a superior performance could be achieved.

Abbreviations: GABP, BP network with genetic algorithm; MSE, mean square error
