# Supplementary material for: Improved Glomerular Filtration Rate Estimation by an Artificial Neural Network
Source: PLoS One. 2013 Mar 13;8(3):e58242. doi: 10.1371/journal.pone.0058242 (PMC3596400; doi:10.1371/journal.pone.0058242)
Supplement: Table S7 — Performance of GABP network with 5 input variables. (DOC) [file pone.0058242.s011.doc]

Table S7. Performance of GABP network with 5 input variables^*^

| Topology | Encoding length | MSE of development data | MSE of internal validation data |
| --- | --- | --- | --- |
| 5-1-1 | 8 | 178.5308 | 177.4366 |
| 5-2-1 | 15 | 173.2786 | 173.5895 |
| 5-3-1 | 22 | 170.1787 | 174.6074 |
| 5-4-1 | 29 | 176.6950 | 167.4387 |
| 5-5-1 | 36 | 165.4390 | 177.8023 |
| 5-6-1 | 43 | 163.7728 | 180.5749 |
| 5-7-1 | 50 | 163.9512 | 174.3362 |

*: When the topology is 5-4-1, a superior performance could be achieved.

Abbreviations: GABP, BP network with genetic algorithm; MSE, mean square error
