# Supplementary material for: Improved Glomerular Filtration Rate Estimation by an Artificial Neural Network
Source: PLoS One. 2013 Mar 13;8(3):e58242. doi: 10.1371/journal.pone.0058242 (PMC3596400; doi:10.1371/journal.pone.0058242)
Supplement: Table S8 — MIV analysis based on GABP network with a topology of 5-4-1. (DOC) [file pone.0058242.s012.doc]

Table S8. MIV analysis based on GABP network with a topology of 5-4-1*

| Input variable | MIV value | Rank |
| --- | --- | --- |
| Serum creatinine | -0.0300 | 1 |
| Age | -0.0142 | 2 |
| Weight | -0.0077 | 3 |
| Serum urea nitrogen | -0.0033 | 4 |
| Height | -0.0003 | 5 |

*: Input variable Height has minimum value of MIV, which indicates Height has the least contribution to estimating dependent variable GFR, so a new GABP network could be constructed without Height.

Abbreviations: GABP, BP network with genetic algorithm; MIV, mean impact value
