# Supplementary material for: Improved Glomerular Filtration Rate Estimation by an Artificial Neural Network
Source: PLoS One. 2013 Mar 13;8(3):e58242. doi: 10.1371/journal.pone.0058242 (PMC3596400; doi:10.1371/journal.pone.0058242)
Supplement: Table S9 — Performance of GABP network with 4 input variables. (DOC) [file pone.0058242.s013.doc]

Table S9. Performance of GABP network with 4 input variables^*^

| Topology | Encoding length | MSE of development data | MSE of internal validation data |
| --- | --- | --- | --- |
| 4-1-1 | 7 | 180.1332 | 176.7971 |
| 4-2-1 | 13 | 177.0907 | 167.9637 |
| 4-3-1 | 19 | 175.3468 | 168.5215 |
| 4-4-1 | 25 | 173.1068 | 169.1010 |
| 4-5-1 | 31 | 172.5527 | 171.2932 |

*: When the topology is 4-2-1, a superior performance could be achieved.

Abbreviations: GABP, BP network with genetic algorithm; MSE, mean square error
