# Supplementary material for: Improved Glomerular Filtration Rate Estimation by an Artificial Neural Network
Source: PLoS One. 2013 Mar 13;8(3):e58242. doi: 10.1371/journal.pone.0058242 (PMC3596400; doi:10.1371/journal.pone.0058242)
Supplement: Table S11 — Performance of GABP network with 3 input variables. (DOC) [file pone.0058242.s015.doc]

Table S11. Performance of GABP network with 3 input variables^*^

| Topology | Encoding length | MSE of development data | MSE of internal validation data |
| --- | --- | --- | --- |
| 3-1-1 | 6 | 179.8319 | 179.2179 |
| 3-2-1 | 13 | 173.9099 | 178.4718 |
| 3-3-1 | 16 | 166.3833 | 173.4562 |
| 3-4-1 | 21 | 173.4528 | 172.4370 |
| 3-5-1 | 26 | 173.2488 | 174.3767 |
| 3-6-1 | 31 | 167.4602 | 178.9914 |

*: When the topology is 3-4-1, a superior performance could be achieved.

Abbreviations: GABP, BP network with genetic algorithm; MSE, mean square error
