# Supplementary material for: Improved Glomerular Filtration Rate Estimation by an Artificial Neural Network
Source: PLoS One. 2013 Mar 13;8(3):e58242. doi: 10.1371/journal.pone.0058242 (PMC3596400; doi:10.1371/journal.pone.0058242)
Supplement: Table S12 — MIV analysis based on GABP network with a topology of 3-4-1. (DOC) [file pone.0058242.s016.doc]

Table S12. MIV analysis based on GABP network with a topology of 3-4-1^*^

| Input variable | MIV value | Rank |
| --- | --- | --- |
| Serum creatinine | -0.0324 | 1 |
| Age | -0.0146 | 2 |
| Weight | -0.0104 | 3 |

*: Input variable Weight has minimum value of MIV, which indicates Weight has the least contribution to estimating dependent variable GFR, so a new GABP network could be constructed without Weight.

Abbreviations: GABP, BP network with genetic algorithm; MIV, mean impact value
