# Supplementary material for: Improved Glomerular Filtration Rate Estimation by an Artificial Neural Network
Source: PLoS One. 2013 Mar 13;8(3):e58242. doi: 10.1371/journal.pone.0058242 (PMC3596400; doi:10.1371/journal.pone.0058242)
Supplement: Table S13 — Performance of GABP network with 2 input variables. (DOC) [file pone.0058242.s017.doc]

Table S13. Performance of GABP network with 2 input variables^*^

| Topology | Encoding length | MSE of development data | MSE of internal validation data |
| --- | --- | --- | --- |
| 2-1-1 | 5 | 181.7323 | 184.6147 |
| 2-2-1 | 9 | 180.3980 | 174.3187 |
| 2-3-1 | 13 | 181.6159 | 172.2004 |
| 2-4-1 | 17 | 176.5798 | 180.7913 |
| 2-5-1 | 21 | 179.8890 | 174.3600 |

*: When the topology is 2-3-1, a superior performance could be achieved.

Abbreviations: GABP, BP network with genetic algorithm; MSE, mean square error
