# Supplementary material for: Improved Glomerular Filtration Rate Estimation by an Artificial Neural Network
Source: PLoS One. 2013 Mar 13;8(3):e58242. doi: 10.1371/journal.pone.0058242 (PMC3596400; doi:10.1371/journal.pone.0058242)
Supplement: Table S14 — MIV analysis based on GABP network with a topology of 2-3-1. (DOC) [file pone.0058242.s018.doc]

Table S14. MIV analysis based on GABP network with a topology of 2-3-1*

| Input variable | MIV^#^ value | Rank |
| --- | --- | --- |
| Serum creatinine | -0.0325 | 1 |
| Age | -0.0143 | 2 |

*: Input variable Age has minimum value of MIV, which indicates Age has the less contribution to estimating dependent variable GFR, so a new GABP network could be constructed without Age.

Abbreviations: GABP, BP network with genetic algorithm; MIV, mean impact value
