# Supplementary material for: Improved Glomerular Filtration Rate Estimation by an Artificial Neural Network
Source: PLoS One. 2013 Mar 13;8(3):e58242. doi: 10.1371/journal.pone.0058242 (PMC3596400; doi:10.1371/journal.pone.0058242)
Supplement: Table S15 — Performance of GABP network with 1 input variable. (DOC) [file pone.0058242.s019.doc]

Table S15. Performance of GABP network with 1 input variable^*^

| Topology | Encoding length | MSE of development data | MSE of internal validation data |
| --- | --- | --- | --- |
| 1-1-1 | 4 | 210.4027 | 213.6814 |
| 1-2-1 | 7 | 211.4018 | 206.9868 |
| 1-3-1 | 10 | 210.5173 | 208.6912 |
| 1-4-1 | 13 | 211.9999 | 206.3892 |
| 1-5-1 | 16 | 211.9065 | 206.1348 |

*: When the topology is 1-5-1, a superior performance could be achieved.

Abbreviations: GABP, BP network with genetic algorithm; MSE, mean square error
