# Supplementary material for: Improved Glomerular Filtration Rate Estimation by an Artificial Neural Network
Source: PLoS One. 2013 Mar 13;8(3):e58242. doi: 10.1371/journal.pone.0058242 (PMC3596400; doi:10.1371/journal.pone.0058242)
Supplement: Table S16 — Overall performance of agreement between eGFR and sGFR in GABP networks with different input variables in the internal validation data set. (DOC) [file pone.0058242.s020.doc]

Table S16. Overall performance of agreement between eGFR and sGFR in GABP networks with different input variables in the internal validation data set

|  | Precision | slope of regression line with the X-axis^*^ (95% CI) | intercept of regression line with the Y-axis^*^ (95% CI) |
| --- | --- | --- | --- |
| GABP7 | 50.8 | -0.10(-0.15，-0.04) | 3.98(0.97，6.98) |
| GABP6 | 51.2 | -0.12(-0.17，-0.06) | 4.83(1.80，7.85) |
| GABP5 | 50.8 | -0.07(-0.13，-0.02) | 3.31(0.30，6.32) |
| GABP4 | 50.9 | -0.11(-0.17，-0.05) | 5.28(2.26，8.30) |
| GABP3 | 51.6 | -0.11(-0.17，-0.05) | 5.40(2.34，8.47) |
| GABP2 | 51.5 | -0.12(-0.18，-0.06) | 5.13(2.09，8.17) |
| GABP1 | 56.4 | -0.16(-0.22，-0.09) | 6.79(3.44，10.14) |

*：*P*＜0.05 compared with GABP7 network-GFR.

Abbreviations: eGFR, estimated glomerular filtration rate; sGFR, standard glomerular filtration rate; GABP: BP network with genetic algorithm
