# Supplementary material for: Improved Glomerular Filtration Rate Estimation by an Artificial Neural Network
Source: PLoS One. 2013 Mar 13;8(3):e58242. doi: 10.1371/journal.pone.0058242 (PMC3596400; doi:10.1371/journal.pone.0058242)
Supplement: Table S17 — Overall performance of difference and accuracy between eGFR and sGFR in GABP networks with different number of input variables in the internal validation data set. (DOC) [file pone.0058242.s021.doc]

Table S17. Overall performance of difference and accuracy between eGFR and sGFR in GABP networks with different number of input variables in the internal validation data set

|  | Median of difference （25%, 75% Percentile） | Median % Absolute difference （25%, 75% Percentile） | Accuracy within | | |
| --- | --- | --- | --- | --- | --- |
|  |  |  | 15% | 30% | 50% |
| GABP7 | 0.09(-7.76，6.81) | 18.66( 8.14，34.79) | 41.3 | 69.1 | 84.0 |
| GABP6 | 0.00(-5.97，6.38) | 17.68( 9.59，32.89) | 42.8 | 71.4 | 85.9 |
| GABP5 | 0.44(-7.28，7.08) | 19.38(9.52，34.88) | 36.4 | 67.7 | 85.1 |
| GABP4 | 1.06(-7.07，7.86) ^*^ | 20.93(9.96，34.88) | 39.8 | 68.0 | 85.5 |
| GABP3 | 0.90(-6.67，7.17) ^*^ | 18.48(9.96，33.37) | 38.7 | 69.5 | 85.5 |
| GABP2 | 0.67(-7.08，7.06) | 19.01(7.89，36.07) | 39.8 | 66.5 | 84.8 |
| GABP1 | 0.99(-7.63，7.76) | 21.78(9.93，40.73) | 35.7 | 65.4 | 84.8 |

*：*P*＜0.05 compared with GABP7 network-GFR.

Abbreviations: eGFR, estimated glomerular filtration rate; sGFR, standard glomerular filtration rate; GABP: BP network with genetic algorithm
