# Supplementary material for: Improved Glomerular Filtration Rate Estimation by an Artificial Neural Network
Source: PLoS One. 2013 Mar 13;8(3):e58242. doi: 10.1371/journal.pone.0058242 (PMC3596400; doi:10.1371/journal.pone.0058242)
Supplement: Table S18 — Performances between eGFR and sGFR in different stages of CKD in the external validation data set. (DOC) [file pone.0058242.s022.doc]

Table S18. Performances between eGFR and sGFR in different stages of CKD in the external validation data set

|  | Median of difference （25%, 75% Percentile） | Median % Absolute （25%, 75% Percentile） | Accuracy within | | |
| --- | --- | --- | --- | --- | --- |
|  |  |  | 15% | 30% | 50% |
| **CKD 1-2 stages** |  |  |  |  |  |
| CG equation | 11.60(-7.13，27.53)^*^ | 20.09(10.22，37.35)^*^ | 39.3^†^ | 68.2^*^ | 84.1^*^ |
| MDRD1 equation | 13.54(-1.18，31.65)^*^ | 21.61(9.41，45.63)^*^ | 38.3^†^ | 61.7^*^ | 79.4^*^ |
| MDRD4 equation | 15.92(2.23，35.89)^*^ | 28.20(9.26，48.60)^*^ | 37.4^†^ | 56.0^*^ | 76.6^*^ |
| CKD-EPI equation | 12.64(-0.02，24.56)^*^ | 19.15(7.66，35.64)^*^ | 43.0^†^ | 68.2^*^ | 91.6^*^ |
| GABP6 network | -8.60(-16.23，0.99) | 13.11(5.93，19.01) | 63.6 | 90.7 | 100.0 |
| **CKD 3 stage** |  |  |  |  |  |
| CG equation | -2.48(-13.90，9.68) | 28.67(11.29，46.74)^*^ | 29.3^*^ | 52.9^*^ | 77.9^*^ |
| MDRD1 equation | -1.49(-16.45，13.70) | 31.85(13.44，52.61)^*^ | 25.7^*^ | 45.0^*^ | 72.9^*^ |
| MDRD4 equation | 0.16(-14.76，13.37) | 31.49(14.37，54.34)^*^ | 25.0^*^ | 46.4^*^ | 72.1^*^ |
| CKD-EPI equation | -1.29(-16.16，12.69) | 30.85(16.49，52.95)^*^ | 23.6^*^ | 48.6^*^ | 71.4^*^ |
| GABP6 network | 0.66(-7.27，6.75) | 16.49(8.87，28.92) | 48.6 | 77.1 | 92.9 |
| **CKD 4-5 stages** |  |  |  |  |  |
| CG equation | -4.07(-7.76，1.98)^*^ | 35.71(19.10，53.54) | 18.6^‡^ | 44.1^‡^ | 70.6 |
| MDRD1 equation | -5.82(-10.02，0.09)^*^ | 42.57(20.40，58.41)^‡^ | 14.7^†^ | 33.0^†^ | 63.7 |
| MDRD4 equation | -5.50(-9.99，1.31)^*^ | 41.88(21.44，57.62)^‡^ | 18.6^‡^ | 35.3^†^ | 62.7^‡^ |
| CKD-EPI equation | -6.65(-10.52，-0.07)^*^ | 43.99(23.28，61.64)^‡^ | 14.7^†^ | 31.4^†^ | 58.8^‡^ |
| GABP6 network | 2.86(-1.53，7.80) | 24.66(11.06，45.46) | 34.3 | 55.9 | 77.5 |

*:*P*＜0.001 compared with GABP6 network-GFR.

†:*P*＜0.01 compared with GABP6 network-GFR.

‡:*P*＜0.05 compared with GABP6 network-GFR.

Abbreviations: CG: Cockcroft-Gault; MDRD: Modification of Diet in Renal Disease; CKD-EPI: Chronic Kidney Disease Epidemiology Collaboration; GABP: BP network with genetic algorithm
