# Supplementary material for: Improved Glomerular Filtration Rate Estimation by an Artificial Neural Network
Source: PLoS One. 2013 Mar 13;8(3):e58242. doi: 10.1371/journal.pone.0058242 (PMC3596400; doi:10.1371/journal.pone.0058242)
Supplement: Table S19 — Classification the CKD stage by the estimation models in different stages of CKD. (DOC) [file pone.0058242.s023.doc]

Table S19. Classification the CKD stage by the estimation models in different stages of CKD

|  | CKD staged by sGFR | | | | |
| --- | --- | --- | --- | --- | --- |
|  | stage 1(n=32) | stage 2(n=75) | stage 3(n=140) | stage 4(n=80) | stage 5(n=22) |
| CKD staged by 6-variable MDRD equation n(%) | | | | | |
| stage 1(n=73) | 29(39.7)^†^ | 36(49.3)^†^ | 8(11.0) | 0(0.0) | 0(0.0) |
| stage 2(n=62) | 3(4.8)^†^ | 31(50.0) | 28(45.2)^*^ | 0(0.0) | 0(0.0) |
| stage 3(n=79) | 0(0.0) | 7(8.9) | 62(78.5) | 8(10.0) | 2(2.5) |
| stage 4(n=61) | 0(0.0) | 1(1.6) | 30(49.2)^†^ | 28(45.9) | 2(3.3) |
| stage 5(n=74) | 0(0.0) | 0(0.0) | 12(16.2) | 44(59.5) | 18(24.3) |
| CKD staged by 4-variable MDRD equation n(%) | | | | | |
| stage 1(n=80) | 29(36.2)^†^ | 40(50.0)^†^ | 11(13.8) | 0(0.0) | 0(0.0) |
| stage 2(n=57) | 3(5.3)^†^ | 27(47.4)^‡^ | 27(47.4)^†^ | 0(0.0) | 0(0.0) |
| stage 3(n=81) | 0(0.0) | 7(8.6) | 65(80.2) | 7(8.6) | 2(2.5) |
| stage 4(n=56) | 0(0.0) | 1(1.8) | 26(46.4)^†^ | 27(48.2)^‡^ | 2(3.6) |
| stage 5(n=75) | 0(0.0) | 0(0.0) | 11(14.7) | 46(61.3) | 18(24.0) |
| CKD staged by Cockcroft-Gault equation n(%) | | | | | |
| stage 1(n=59) | 25(42.4)^†^ | 29(49.2)^†^ | 5(8.5) | 0(0.0) | 0(0.0) |
| stage 2(n=68) | 6(8.8)^†^ | 35(51.5) | 27(39.7)^†^ | 0(0.0)[0.0] | 0(0.0) |
| stage 3(n=93) | 1(1.1) | 10(10.8) | 71(76.3) | 11(11.8) | 0(0.0) |
| stage 4(n=73) | 0(0.0) | 1(1.4) | 31(42.5)^†^ | 36(49.3) | 5(6.8) |
| stage 5(n=56) | 0(0.0) | 0(0.0) | 6(10.7) | 33(58.9) | 17(30.4) |
| CKD staged by CKD-EPI equation n(%) | | | | | |
| stage 1(n=80) | 29(36.3)^†^ | 41(51.2)^†^ | 10(12.5) | 0(0.0) | 0(0.0) |
| stage 2(n=58) | 3(5.2)^†^ | 27(46.6) | 28(48.3)^*^ | 0(0.0) | 0(0.0) |
| stage 3(n=76) | 0(0.0) | 6(7.9) | 62(81.6) | 7(9.2) | 1(1.3) |
| stage 4(n=58) | 0(0.0) | 1(1.7) | 29(50.0)^†^ | 26(44.8)^‡^ | 2(3.4) |
| stage 5(n=77) | 0(0.0) | 0(0.0) | 11(14.3) | 47(61.0) | 19(24.7) |
| CKD staged by GABP 6 network n(%) | | | | | |
| stage 1(n=11) | 10(90.9) | 1(9.1) | 0(0.0) | 0(0.0) | 0(0.0) |
| stage 2(n=103) | 22(21.4) | 63(61.2) | 18(17.5) | 0(0.0) | 0(0.0) |
| stage 3(n=133) | 0(0.0) | 11(8.3) | 103(77.4) | 18(13.5) | 1(0.8) |
| stage 4(n=76) | 0(0.0) | 0(0.0) | 17(22.4) | 49(64.5) | 10(13.2) |
| stage 5(n=26) | 0(0.0) | 0(0.0) | 2(7.7) | 13(50.0) | 11(42.3) |

*:*P*＜0.001 compared with GABP6 network-GFR.

†:*P*＜0.01 compared with GABP6 network-GFR.

‡:*P*＜0.05 compared with GABP6 network-GFR.

Abbreviations: CKD, chronic kidney disease; sGFR, standard golmerular filtration rate; MDRD: Modification of Diet in Renal Disease; CKD-EPI: Chronic Kidney Disease Epidemiology Collaboration; GABP: BP network with genetic algorithm
